# Invariant Natural Killer T Cells as Key Players in Host Resistance against *Paracoccidioides brasiliensis*

**DOI:** 10.1155/2021/6673722

**Published:** 2021-04-16

**Authors:** Joes Nogueira-Neto, Flavio V. Loures, Alessandra S. Schanoski, David A. G. Andrade, Michelangelo B. Gonzatti, Tania A. Costa, Bruno C. Vivanco, Patrícia Xander, Daniela S. Rosa, Vera L. G. Calich, Alexandre C. Keller

**Affiliations:** ^1^Departamento de Microbiologia, Imunologia e Parasitologia, Escola Paulista de Medicina, Universidade Federal de São Paulo, Brazil; ^2^Departamento de Imunologia, Instituto de Ciências Biomédicas, Universidade de São Paulo, Brazil; ^3^Instituto de Ciência e Tecnologia, Universidade Federal de São Paulo, Campus São José dos Campos, Brazil; ^4^Departamento de Ciências Farmacêuticas, Instituto de Ciências Ambientais, Químicas e Farmacêuticas, Universidade Federal de São Paulo, Campus Diadema, Brazil

## Abstract

Invariant Natural Killer T (iNKT) cells are key players in the immunity to several pathogens; however, their involvement in the resistance to *Paracoccidioides brasiliensis* infection remains unknown. Using splenocytes from CD1d (CD1d^−/−^) and iNKT-deficient (J*α*18^−/−^) mice, we found that iNKT cells are the innate source of IFN-*γ* after *P. brasiliensis* infection and are required to potentiate macrophage oxidative burst and control fungal growth. To determine whether iNKT cells contribute *in vivo* to host resistance against *P. brasiliensis* infection, we infected intratracheally wild-type and J*α*18^−/−^ C57BL/6 mouse strains with the virulent Pb18 isolate. iNKT cell deficiency impaired the airway acute inflammatory response, resulting in decreased airway neutrophilia and reduced IFN-*γ*, KC, and nitric oxide (NO) production. The deficient innate immune response of J*α*18^−/−^ mice to Pb18 infection resulted in increased fungal burden in the lungs and spleen. Besides, the activation of iNKT cells *in vivo* by administration of the exogenous iNKT ligand *α*-galactosylceramide (*α*-GalCer) improved host resistance to *P. brasiliensis* infection. Although the mechanisms responsible for this phenomenon remain to be clarified, *α*-GalCer treatment boosted the local inflammatory response and reduced pulmonary fungal burden. In conclusion, our study is the first evidence that iNKT cells are important for the protective immunity to *P. brasiliensis* infection and their activation by an exogenous ligand is sufficient to improve the host resistance to this fungal infection.

## 1. Introduction

Paracoccidioidomycosis (PCM) is caused by a fungus from the *Paracoccidioides* genus and is considered one of the highest causes of mortality among Brazilian systemic mycoses [1]. Clinical studies demonstrated a relationship between the characteristics of the immune response and disease severity [2]. In humans, a prominent Th1 response is associated with infection without disease, the chronic form of the disease with Th1/Th17 immunity, and the most severe manifestation, the acute or juvenile form, shows a prominent Th2/Th9 profile [1]. These data are supported by murine models of *P. brasiliensis* infection showing the association between the classical Th1 immune response, with high levels of IL-2 and IFN-*γ*, with resistance against the fungi [3, 4].

The lung is the primary site of fungal infection, where alveolar macrophages (M*Φ*) recognize Pathogen-Associated Molecular Patterns (PAMPs) through several Pattern Recognition Receptors (PRRs) [5]. Although *P. brasiliensis* proliferates in resident alveolar M*Φ*, these cells can inhibit fungal growth upon activation mediated by the synergistic action between lytic enzymes and metabolites from the oxidative burst that mediate fungal killing [6, 7]. In parallel, these cells potentiate the immunity against the fungi by secreting cytokines and chemokines that coordinate the influx and activation of other immune cells, such as neutrophils and T lymphocytes, to the site of infection [8]. In this scenario, Th1-biased lymphocytes increase the fungicidal ability of phagocytes, promoting resistance against the pathogen [9, 10]. Although IFN-*γ* drives the quality of the inflammatory response during the acute phase of *P. brasiliensis* infection, the source of the early IFN-*γ* production remains unclear. Because invariant Natural Killer T (iNKT) lymphocytes are poised for the rapid production of IFN-*γ*, we hypothesized that they are important players in the immunity to *P. brasiliensis* infection [11].

The iNKT cells are a subpopulation of unconventional T lymphocytes that due to an invariant T cell receptor (TCR) promptly respond to lipid antigens presented in the context of CD1d molecules [12, 13]. In addition to this unique specificity, they can rapidly secrete several cytokines and chemokines, acting as a bridge between innate and adaptive immunity [14, 15]. This ability confers an essential immune regulatory function to these cells that participate in diverse types of immune responses, including those against pathogens [16, 17]. Although a previous study described that iNKT cells from both healthy controls and cured PCM patients have the same ability to expand and produce cytokines, there are no data regarding their role in *in vivo* models of *P. brasiliensis* infection [18]. Therefore, we used the intratracheal model of *P. brasiliensis* infection with the virulent Pb18 strain, and wild-type (WT) and iNKT-deficient (J*α*18^−/−^) C57BL-6 mice to determine the role of these cells in host resistance to *P. brasiliensis* [13, 19].

Our findings show that iNKT lymphocytes are the primary innate source of IFN-*γ*, and their deficiency leads to impaired airway inflammation and increased pulmonary fungal burden. Furthermore, the specific activation of iNKT cells by *α*-galactosylceramide (*α*-GalCer) in an ongoing disease increased host resistance against this fungal pathogen. Therefore, iNKT cells were shown to play an essential role in shaping the protective immunity against the fungi, and the treatment with specific iNKT agonists potentiates host resistance to PCM.

## 2. Materials and Methods

### 2.1. Animals

Isogenic male C57Bl/6 mice from wild-type (WT), CD1d^−/−^, and iNKT-deficient strain (J*α*18^−/−^), aged 8–12 weeks, were obtained from the Animal Care Facility at the Federal University of São Paulo (UNIFESP). The C57Bl/6 J*α*18^−/−^ strain was a gift from Dr. Masaru Taniguchi at the RIKEN Research Center for Allergy and Immunology (Japan) [13]. All animals were housed in individual standard cages and had free access to food and water. All procedures were previously reviewed and approved by the internal ethics committee of Universidade de São Paulo (USP-180/2011) and Universidade Federal de São Paulo (UNIFESP–CEP 0372/12).

### 2.2. Fungus

The virulent isolate Pb18 from *Paracoccidioides brasiliensis* was used throughout the experiments outlined in this work [20]. Pb18 yeast cells were subcultivated every seven days in semisolid Fava-Netto culture medium at 37°C until use. The yeast cells were collected and washed with sterile phosphate-buffered saline (PBS, pH 7.2). Fungal viability was determined by the Janus Green B vital dye. All experimental procedures were carried out with fungal suspensions presenting viability between 90 and 95%.

### 2.3. Peritoneal M*Φ*, Splenocytes, and *In Vitro* Culture

A sterile solution of 3% thioglycolate was injected in the peritoneal cavity, and four days later, peritoneal leukocytes were collected, and thioglycolate-elicited peritoneal M*Φ* were isolated by adherence (2 h at 37°C in 5% CO_2_) in plastic-bottom tissue-culture plates. Spleens were homogenized using the plunger end of a 3 mL syringe and a 70 *μ*m strainer, erythrocytes were removed using ACK solution (0.15 M NH_4_Cl; 1 mM KHCO_3_; 0.1 mM Na_2_ EDTA), and cell viability was determined using trypan blue. After removing nonadherent cells, M*Φ* were cultivated alone or with *P. brasiliensis* yeasts in an M*Φ*: yeast ratio of 10 : 1 (2 h at 37°C in 5% CO_2_) to allow fungi adhesion and ingestion. Supernatants were removed, and cells were gently washed with PBS to remove any noningested or nonadherent yeast. After that, splenocytes from WT, CD1d^−/−^ or J*α*18^−/−^ mice were added to the culture in an M*Φ*: splenocyte ratio of 1 : 10. After 48 h of coculture, supernatants were collected and analyzed for IFN-*γ*, IL-10, and NO production. After removing coculture supernatants, the wells were washed with distilled water to lyse the cells, and suspensions were collected in individual tubes. Cell homogenates were assayed for the presence of viable yeasts, as previously described [21].

### 2.4. Colony-Forming Units Assay

The number of viable Pb18 yeasts in cell cultures and infected organs was determined by counting the number of colony-forming units (CFU) as previously described [21].

### 2.5. Induction of Experimental PCM

Animals were deeply anesthetized and infected by intratracheal (i.t.) delivery of 1 x 10^6^ viable Pb yeast cells. Animals were euthanized 72 hours postinfection to assess iNKT cells' role in the acute phase of infection. Depending on the objective of the experiment, euthanasia occurred forty-five days or eight weeks postinfection to determine the severity of Pb infection.

### 2.6. Bronchoalveolar Lavage Fluid (BAL)

To determine the content of inflammatory cells in the airways, mice were euthanatized, the trachea was surgically exposed, and 0.5 mL of cold PBS was injected with a plastic cannula to obtain the BAL. After BAL collection, the total number of cells was determined in the Neubauer chamber and samples were centrifuged for supernatant analysis. Cellular precipitated was suspended in PBS for cytocentrifugation (Shandon, USA or Fanem, Br), and cellular populations were analyzed upon slides staining with hematoxylin/eosin.

### 2.7. Cytokine and Chemokine Production

IFN-*γ* and KC levels in BAL were analyzed with a multiplex kit (Millipore, USA) following the manufacturer's recommendations. The IFN-*γ* levels in culture supernatants were quantified using ELISA (R&D Systems, USA).

### 2.8. Nitric Oxide Production

Nitric oxide production in BAL was assessed using Nitrate/Nitrite Colorimetric Assay (Cayman Chemicals, USA) according to the manufacturer's recommendations. In culture supernatants, NO production was quantified by nitrite accumulation in the supernatants using a standard Griess reaction [22].

### 2.9. Flow Cytometry Assay

To determine the inflammatory state of lung parenchyma, the organs were digested with a DNAse (1 mg/mL) and collagenase (2 mg/mL) solution (Invitrogen), homogenized, centrifuged in Percoll 35% (G&E, USA) solution, and stained for different surface markers (eBioscience, USA). The T lymphocyte population was analyzed according to the expression of CD3, CD4, CD8, and CD69. Myeloid-derived cells were analyzed according to the expression of GR1, CD11b, and MHC-II. All data concerning the FACS assays were analyzed using the FlowJo software (BD, USA), according to specific cell population characteristics. Further information about analysis strategy is presented in Supplementary Figures 1 and 2.

### 2.10. *α*-Galactosylceramide Treatment

At the week 4 after infection, mice were treated intravenously (i.v.) with 10 *μ*g of the NKT cell agonist *α*-GalCer (KRN7000, Cayman Chemical Company, USA) and euthanized four weeks later to determine the fungal burden in the lung.

### 2.11. Statistical Analysis

For the comparison between two groups, we used a 2-tailed unpaired *t*-test, and for multiple comparisons, we used a two-way ANOVA followed by Turkey's or Bonferroni's multiple comparison test. All statistical analyses were performed using the GraphPad Prism 7 software (San Diego, CA).

## 3. Results

### 3.1. Invariant Natural Killer T Cells Are the Innate Source of IFN-*γ* in Response to Fungal Infection and Are Required to Control *P. brasiliensis* Growth

**Figure 1:**
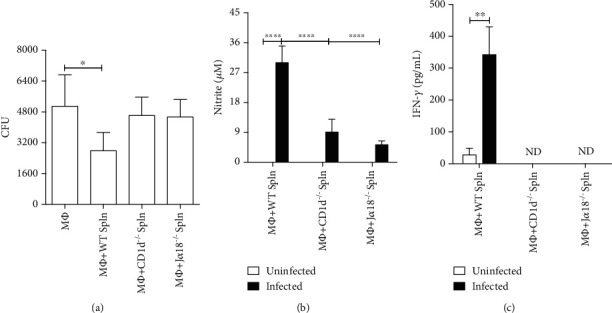
The invariant Natural Killer T cells drive *P. brasiliensis* killing by macrophages and respond for the innate source of IFN-*γ* upon fungal infection. Thioglycolate-elicited peritoneal M*Φ* from C57Bl/6 mice were infected with Pb18 yeast (1 : 10) for 2 h. After washing, splenocytes (Spln) from WT, CD1d^−/−^, or J*α*18^−/−^ C57BL/6 mouse strains were added to the M*Φ* culture (10 : 1) for 48 h. After this period, supernatant was collected, and adherent cells were lysed with distilled water for fungal recovery. (a) Number of viable cell yeast obtained by colony-forming units assay (CFU). (b) NO levels in culture supernatants. (c) IFN-*γ* levels in the culture supernatants (ND = not detected). Data represent the mean ± SD of quintuplicate samples from 1 of 2 independent experiments (a, b) and of 1 independent experiment (c). ^∗^*p* < 0.05; ^∗∗^*p* < 0.005; ^∗∗∗∗^*p* < 0.0001.

To determine the role of the iNKT cells in the immunity against *P. brasiliensis*, we tested the ability of naïve splenocytes from CD1d^−/−^ mouse, deficient in diverse CD1d-restricted cells, or J*α*18^−/−^ mouse strain, which lacks only the CD1d-restrict iNKT cell subset, to respond to fungal infection [13, 23]. Thioglycolate-elicited peritoneal M*Φ* were exposed to Pb18 yeasts for 2 h, followed by coculture with 5 x 10^6^ splenocytes from naive WT, CD1d^−/−^, or J*α*18^−/−^ mice. Two days later, culture supernatants were collected, and M*Φ* were lysed to determine the number of viable yeasts.  Figure 1(a) demonstrates that the addition of naive WT splenocytes to M*Φ* culture increased the fungicidal activity of macrophages. In contrast, splenocytes from both CD1d^−/−^ and J*α*18^−/−^ mice failed to increase M*Φ* killing capacity, indicating that the ability of naive splenocytes to control fungal growth depends on iNKT cells. In concordance, coculture with splenocytes from WT mice resulted in a higher NO production than those from CD1d^−/−^ and J*α*18^−/−^ (Figure 1(b)). Further analysis revealed that splenocytes from both CD1d^−/−^ and J*α*18^−/−^ failed to produce IFN-*γ* in response to fungal infection (Figure 1(c)). Therefore, these data show that among CD1d-restrict cells, the iNKT cell subset is the primary source of IFN-*γ* during *P. brasiliensis* infection and essential for early control of fungal growth.

### 3.2. Deficiency in iNKT Cells Impairs the Acute Airway Inflammation upon *P. brasiliensis* Infection

**Figure 2:**
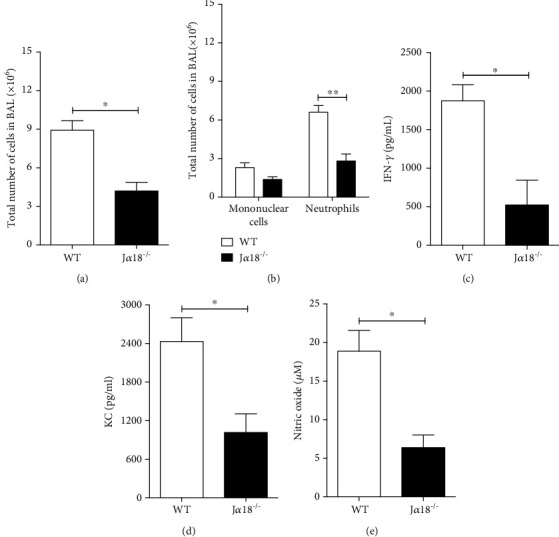
Deficiency in invariant Natural Killer T lymphocytes impairs the acute inflammatory response against *P. brasiliensis* infection. WT and J*α*18^−/−^ C57BL/6 mouse strains were infected with 1 x 10^6^ Pb18 yeasts, and 72 h later, the BAL content was analyzed for: (a) total number of cells; (b) presence of mononuclear cells and neutrophils; BAL levels of (c) IFN-*γ*, (d) KC, and (e) NO. Data represent the mean ± SD from 1 of 2 independent experiments (*n* = 4 − 5/group). ^∗^*p* < 0.05; ^∗∗^*p* < 0.005.

To determine the contribution of iNKT cells to the innate immune response *against P. brasiliensis* infection, we infected WT or J*α*18^−/−^ C57Bl/6 mice by intratracheal (i.t.) route with 1 x 10^6^ living Pb18 yeast cells, and the BAL was collected 72 h later. J*α*18^−/−^ mice presented a lower number of cells in the BAL compared to WT animals, which reflected an impaired airway neutrophilia (Figures 2(a) and 2(b), respectively). In accordance, the absence of iNKT cells resulted in lower levels of proinflammatory cytokines and chemokines, such as IFN-*γ* and KC in the airways (Figures 2(c) and 2(d), respectively). Moreover, NO levels in BAL were lower in the J*α*18^−/−^ animals than in the WT group (Figure 2(e)). Therefore, these data demonstrate that iNKT cells play an important role in the acute phase of the inflammatory response following pulmonary Pb18 infection.

### 3.3. iNKT Cells Drive Early CD8 T Cell Activation and Influence the Presence of Distinct GR1^+^CD11b^+^ Cellular Population in the Lungs of Infected Mice

**Figure 3:**
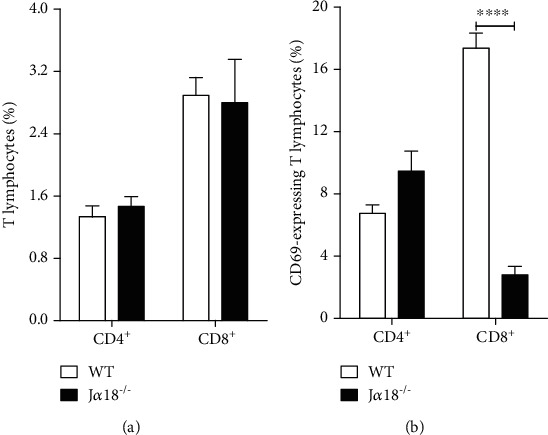
Deficiency in invariant Natural Killer T cells impairs early CD8 T lymphocyte activation during *P. brasiliensis* infection. WT and J*α*18^−/−^ C57BL/6 mouse strains were infected with 1 x 10^6^ Pb18 yeasts, and 72 h later, the lung parenchyma was analyzed for: (a) frequency of CD4^+^ and CD8^+^ T lymphocytes and (b) frequency of CD69-expressing CD4 and CD8 T cells. Data represent the mean ± SD from 1 of 2 independent experiments (*n* = 5/group). ^∗∗∗∗^*p* < 0.0001.

The flow cytometric analysis of lung parenchyma did not reveal any significant difference in the frequency of CD^4^ and CD^8^ T lymphocytes between WT and J*α*18^−/−^ mice (Figure 3(a)). However, the frequency of CD8^+^CD69^+^ T cells in the J*α*18^−/−^ was lower than observed in WT group (Figure 3(b)). Because CD69 molecule represents an early activation marker, this result indicates that iNKT cells are required for the early activation of CD8^+^ T cells [24]. No significant difference was observed regarding CD69 expression in the CD4^+^ subset.

**Figure 4:**
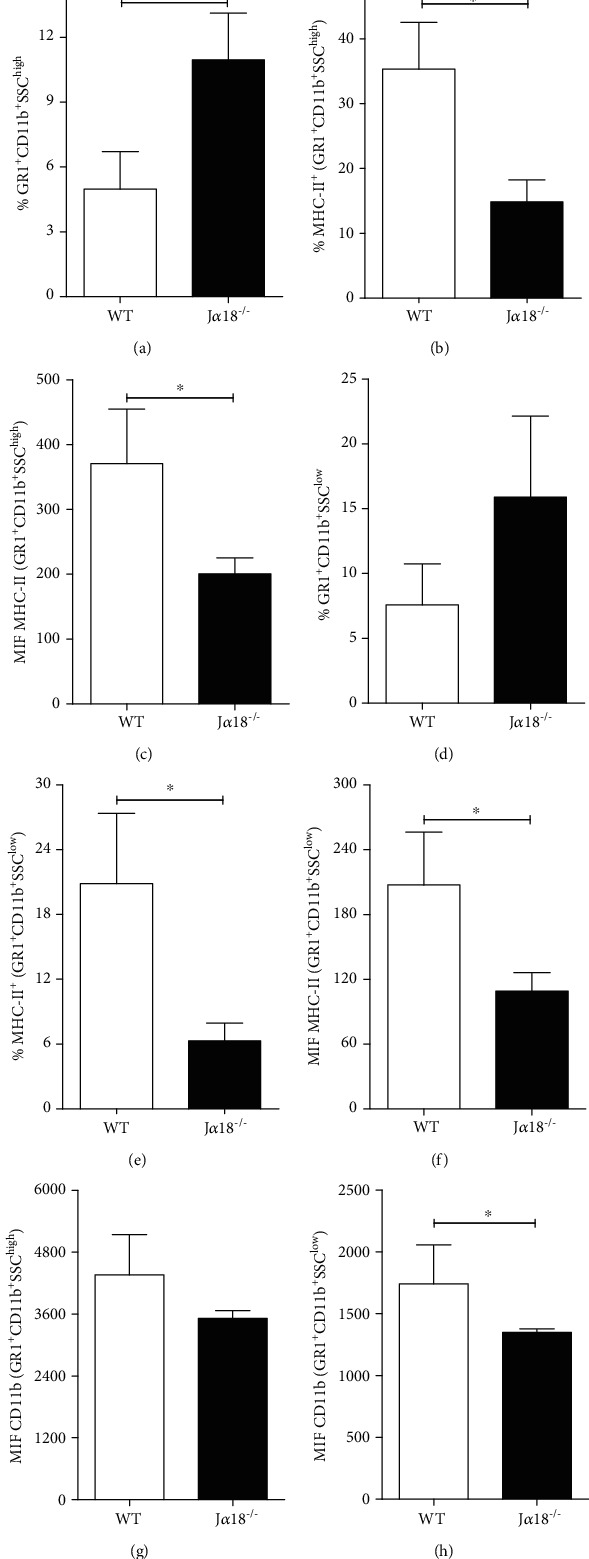
iNKT cells deficiency is associated with the accumulation of suppressor myeloid-derived cells in the lungs of *P. brasiliensis* infected mice. WT and J*α*18^−/−^ C57BL/6 mouse strains were infected with 1 x 10^6^ Pb18 yeasts, and 72 h later, the lung parenchyma was analyzed for: Frequency of (a) GR1^+^CD11b^+^SCC^high^ and (b) GR1^+^CD11b^+^SCC^low^ myeloid-derived cells. Frequency of MHC-II-expressing cells within (b) GR1^+^CD11b^+^SCC^high^ and (e) GR1^+^CD11b^+^SCC^low^ subsets and MHC-II surface expression level (c, f, respectively). Expression levels of CD11b molecules within the (g) GR1^+^CD11b^+^SCC^high^ and (h) GR1^+^CD11b^+^SCC^low^ myeloid-derived cells subsets. Data represent the mean ± SD from 1 of 2 independent experiments (*n* = 5/group). ^∗^*p* < 0.05.

Further analysis revealed the presence of distinct GR1^+^CD11b^+^SSC^high^ and GR1^+^CD11b^+^SSC^low^ cellular populations between WT and J*α*18^−/−^ animals (Figure 4). We first found that the GR1^+^CD11b^+^SSC^high^, but not the GR1^+^CD11b^+^SSC^low^, subset was significantly increased in the J*α*18^−/−^ group versus WT animals (Figures 4(a) and 4(d), respectively). These cellular populations have been described as suppressor or effector myeloid cells according to their functional state [25]. Therefore, to better characterize their immunological status, we first determined the surface expression of the MHC-II molecule [26]. In the WT group, the frequency of MHC-II-expressing cells and its surface expression was higher in both GR1^+^CD11b^+^SSC^high^ and GR1^+^CD11b^+^SSC^low^ cells indicating a proinflammatory profile (Figures 4(b), 4(c), 4(e), and 4(f), respectively) [26]. This notion is corroborated by a higher surface expression of CD11b molecule, especially within the GR1^+^CD11b^+^SSC^low^ subset (Figures 4(g) and 4(h)) [27].

### 3.4. Deficiency in iNKT Cells Impairs Host Resistance against *P. brasiliensis* Infection

**Figure 5:**
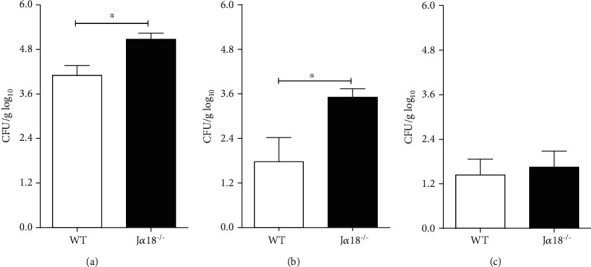
Invariant Natural Killer T cells restrain *P. brasiliensis* growth and dissemination. WT and J*α*18^−/−^ C57BL/6 mouse strains were infected with 1 x 10^6^ Pb18 yeast, and 45 days later, the fungal loads were determined in the lungs (a), spleen (b), and liver (c). Data represent the mean ± SD from 1 of 2 independent experiments (*n* = 5/group). ^∗^*p* < 0.05.

Because iNKT deficiency impaired the early immune response against Pb18, we next determined their influence on a late phase of fungal infection. To this end, WT and J*α*18^−/−^ mice were infected with 1 x 10^6^ living Pb yeast cells, and 45 days later, the lungs, spleen, and liver were analyzed to determine fungal loads. Figures 5(a) and 5(b) demonstrate that J*α*18^−/−^ mice presented a higher number of viable fungal cells in the lungs and spleen than WT animals. No differences in CFUs were detected in the livers (Figure 5(c)). These data indicate that the absence of iNKT cells increases the susceptibility of iNKT-deficient mice to Pb18 infection and support the idea that iNKT cells are important to control fungal growth at the site of infection and its dissemination to other organ.

### 3.5. Treatment of Mice with an iNKT Cells Agonist (*α*-GalCer) Confers Resistance to *P. brasiliensis* Infection

It is well described that activation of iNKT cells by exogenous ligands, especially *α*-GalCer, modulates different immune responses [28]. Thus, C57Bl/6 WT mice were treated with 10 *μ*g of *α*-GalCer at week four after infection to test its effect on the host resistance to *P. brasiliensis* infection. Briefly, four weeks after i.t. infection with 1 x 10^6^ living yeasts, infected mice received one single i.v. injection of 10 *μ*g of *α*-GalCer, and four weeks later, the influx of leukocytes to the airways and pulmonary fungal loads was determined.

**Figure 6:**
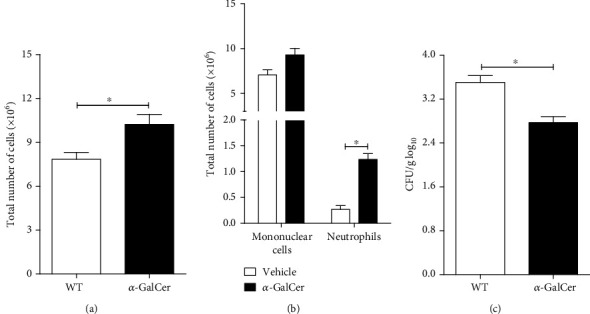
Treatment of *P. brasiliensis* infected mice with *α*-GalCer, an iNKT specific agonist, enhances host resistance to *P. brasiliensis* infection. WT C57BL/6 mice were infected with 1 x 10^6^ Pb18 yeasts and treated four weeks later with 10 *μ*g of *α*-GalCer by the i.v. route. At week 8 after infection, the number of inflammatory cells in the BAL and the number of viable yeasts in the lung parenchyma of *α*-GalCer-treated and untreated mice were determined. (a) Total number of inflammatory cells in BAL. (b) Mononuclear and neutrophil cell counts in the airways. (c) Fungal loads in lung parenchyma. Data represent the mean ± SD from 1 independent experiment (*n* = 5/group). ^∗^*p* < 0.05.

The *α*-GalCer treatment resulted in a more intense inflammatory reaction against *P. brasiliensis* inoculation. The total number of cells and number of neutrophils were higher in the BAL of *α*-GalCer-treated mice compared to nontreated mice (Figures 6(a) and 6(b), respectively). This increased inflammatory response was concomitant with lower fungal loads in the lungs of *α*-GalCer-treated mice (Figure 6(c)). Therefore, these data further indicate the protective role of iNKT cells and suggest that iNKT activation by specific agonist can be used as a possible therapeutic tool in pulmonary paracoccidioidomycosis.

## 4. Discussion

*Paracoccidioides brasiliensis* is an opportunistic fungus, which causes occasional, self-limited, infections in the majority of immunocompetent individuals. However, systemic spreading with severe clinical manifestations has been correlated with intrinsic genetic characteristics of hosts, such as primary immunodeficiencies and individual lifestyle such as smoking, alcohol abuse, malnutrition, and treatment with immunosuppressive drugs [29, 30]. Despite the extensive literature about the importance of innate immunity in the acute host response to infection and fungal control, the role of iNKT cells remains unknown [31]. To determine the impact of invariant Natural Killer T cells in *P. brasiliensis* infection, we used animals that lack the CD1d molecule, which are deficient in diverse CD1d-restricted cells, or the J*α*281 TCR gene segment (J*α*18^−/−^), which results in the specific depletion of the iNKT phenotype [13, 23].

In response to the contact with Pb-infected M*Φ*, naive splenocytes from WT mice produced high levels of IFN-*γ*, induced high levels of NO, and controlled fungal growth. These findings corroborate the notion that *in vitro* control of fungal growth by macrophages correlates with the ability of splenocytes to produce IFN-*γ* [32]. In contrast, splenic cells from CD1d^−/−^ mice failed to produce IFN-*γ*, stimulate NO production, and control *P. brasiliensis* growth. Therefore, we could demonstrate that CD1d-restricted cells are responsible for the splenocytes IFN-*γ* production in response to *P. brasiliensis* infection and exerted an essential role for the early contention of fungal growth.

Several studies demonstrated that CD1d-restricted T cells comprise distinct cell subpopulations [23]. Thus, to demonstrate that it was specifically the iNKT cell subset, the one that exerted a regulatory role in pulmonary PCM, we used the J*α*18^−/−^ mice strain, which lacks these cells due to the deletion of the J*α*281 gene segment [13]. Similar to CD1d^−/−^ splenocytes, naive cells from J*α*18^−/−^ mice failed to produce IFN-*γ*, induce NO production, and control fungal growth. Because no IFN-*γ* production was detected in the presence of naive splenocytes from both CD1d^−/−^ or J*α*18^−/−^ mice, it is conceivable to assume that the iNKT cells are responsible for the IFN-*γ* secretion and the control of fungal growth by *P. brasiliensis* infected M*Φ*.

To determine the *in vivo* influence of iNKT cells on host response to *P. brasiliensis*, we took advantage of the murine model of intratracheal fungal infection. The BAL analysis revealed that during the early phase of infection, the absence of iNKT cells impaired the migration of inflammatory cells to the airways, as determined by reduced airway neutrophilia. In parallel to this finding, iNKT cell deficiency also impaired KC production, a chemokine extensively described as a homing factor for neutrophils [33]. Although the mechanisms that control KC and neutrophilic inflammation are complex, it has been described that during *P. brasiliensis* infection, IFN-*γ* mediates diverse chemokines production, including KC [34]. Thus, it is pertinent to suppose that the lower levels of KC found in the BAL of J*α*18^−/−^-infected mice result from impaired IFN-*γ* production in the absence of iNKT cells. Although iNKT cells are rapid IFN-*γ* producing cells, neutrophils also seem to contribute for IFN-*γ* production during *P. brasiliensis* infection [35]. Thus, the reduced IFN-*γ* levels in J*α*18^−/−^ mice during the acute responses may result from the absence of iNKT cells and the consequent impairment of neutrophil recruitment. The reduced ability to produce IFN-*γ* also impacted the secretion of fungicide, or fungistatic metabolites, such as nitric oxide (NO) [36].

The lung parenchyma analysis confirmed the *in vivo* inability of J*α*18^−/−^ mice to mount a protective inflammatory response. During the acute phase of the infection, iNKT deficiency was associated with a lower frequency of CD69-expressing CD8^+^, but not CD4^+^, T cells indicating their suboptimal activation. Although further experiments are required to better explore the relationship between iNKT and CD8^+^ cells during the acute phase of P*. brasiliensis* infection, the cross-talk between iNKT cells and CD8^+^ T lymphocytes is a phenomenon well recognized. The generation of short-lived effector cells in peripheral lymphoid organs depends on an initial short-lived interaction (0-6 h) followed by a prolonged-lasting contact (12-24 h) [37]. Also, IFN-*γ* production by iNKT cells is essential for the physiological induction of specific CD8 effector T cells [38]. Because the control of fungal loads has been associated with the CD8^+^ T lymphocyte subset, it is possible to propose that iNKT cells play an important role in generating effector CD8T^+^ cells during the beginning of *P. brasiliensis* infection [39, 40]. Furthermore, iNKT cell deficiency was also associated with the accumulation of myeloid-derived suppressor- (MDSC-) like cells. Although the interaction between iNKT lymphocytes and MDSC during infection remains unclear, there is evidence that activated iNKT cells can inhibit the immunosuppressive activity of MDSC [41, 42]. Myeloid cells expressing concomitantly the GR-1 and the CD11b markers represent a group of diverse polymorphonuclear (SSC^high^) and monocytic (SSC^low^) suppressor or effector cells [25, 26, 43]. Although a clear distinction between the suppressor or effector subsets requires very specific assays, the high expression of MHC-II and CD11b molecules may be used to distinguish between these cellular subsets [26, 27]. In this context, we found that during *P. brasiliensis* infection, the frequency of GR-1^+^CD11b^+^ cells expressing low MHC-II and CD11b levels increased in the J*α*18^−/−^ animals, suggesting the appearance of the suppressive subset. Thus, it is reasonable to hypothesize that the early activation of iNKT cells in response to *P. brasiliensis* modulates the appearance of MDSC in the pulmonary environment.

The results discussed above support the idea that the absence of iNKT lymphocytes leads to an immunocompromised microenvironment that impairs host resistance against *P. brasiliensis* infection. Indeed, the importance of iNKT cells in the immunity against *P. brasiliensis* is evident when comparing fungal burdens between WT and J*α*18^−/−^ mice. iNKT cell deficiency resulted in higher fungal loads in both lungs and spleen compared to the WT mice, indicating that these cells are key players in the control of fungus dissemination.

Finally, a previous study demonstrated that a single high dose of *α*-GalCer improved the outcome of *Mycobacterium tuberculosis* infection [44]. Thus, we next addressed the impact of *α*-GalCer administration on *P. brasiliensis*-infected mice. Animals treated with *α*-GalCer four weeks after fungal infection exhibited a significant reduction in the pulmonary fungal loads at week 8 after infection. Although the mechanisms involved in this phenomenon remain an object of study, fungal growth control was parallel to a more robust airway inflammatory response.

In conclusion, this study provides the first evidence of the contribution of iNKT cells to host resistance against *P. brasiliensis* infection and that their activation by exogenous agonists could be considered a therapeutic tool to improve immunity to infection.

## Data Availability

Data is available under reasonable request.

## Supplementary Materials

Supplementary Materials Supplemental Figure 1: analysis of CD4 and CD8 T lymphocytes in lung parenchyma. After exclusion of dead cells, the CD3^+^ cells were analyzed for CD4 or CD8 expression and the frequency of CD69-expressing cells was determined within these populations. Supplemental Figure 2: analysis of GR-1^+^CD11b^+^ subsets in lung parenchyma. After exclusion of dead cells, the GR-1^+^ cells, within the CD11b^+^ subset, were analyzed according to SSC size (high or low). Next, both subsets were analyzed for MHC-II and CD11b expression.
