# Supplementary material for: Invariant Natural Killer T Cells as Key Players in Host Resistance against Paracoccidioides brasiliensis
Source: J Immunol Res. 2021 Apr 16;2021:6673722. doi: 10.1155/2021/6673722 (PMC8064773; doi:10.1155/2021/6673722)

Supplemental Figure 1. Analysis of CD4 and CD8 T lymphocytes in lung parenchyma. After exclusion of dead cells, the CD3$^+$ cells were analyzed for CD4 or CD8 expression and the frequency of CD69-expressing cells was determined within these populations.

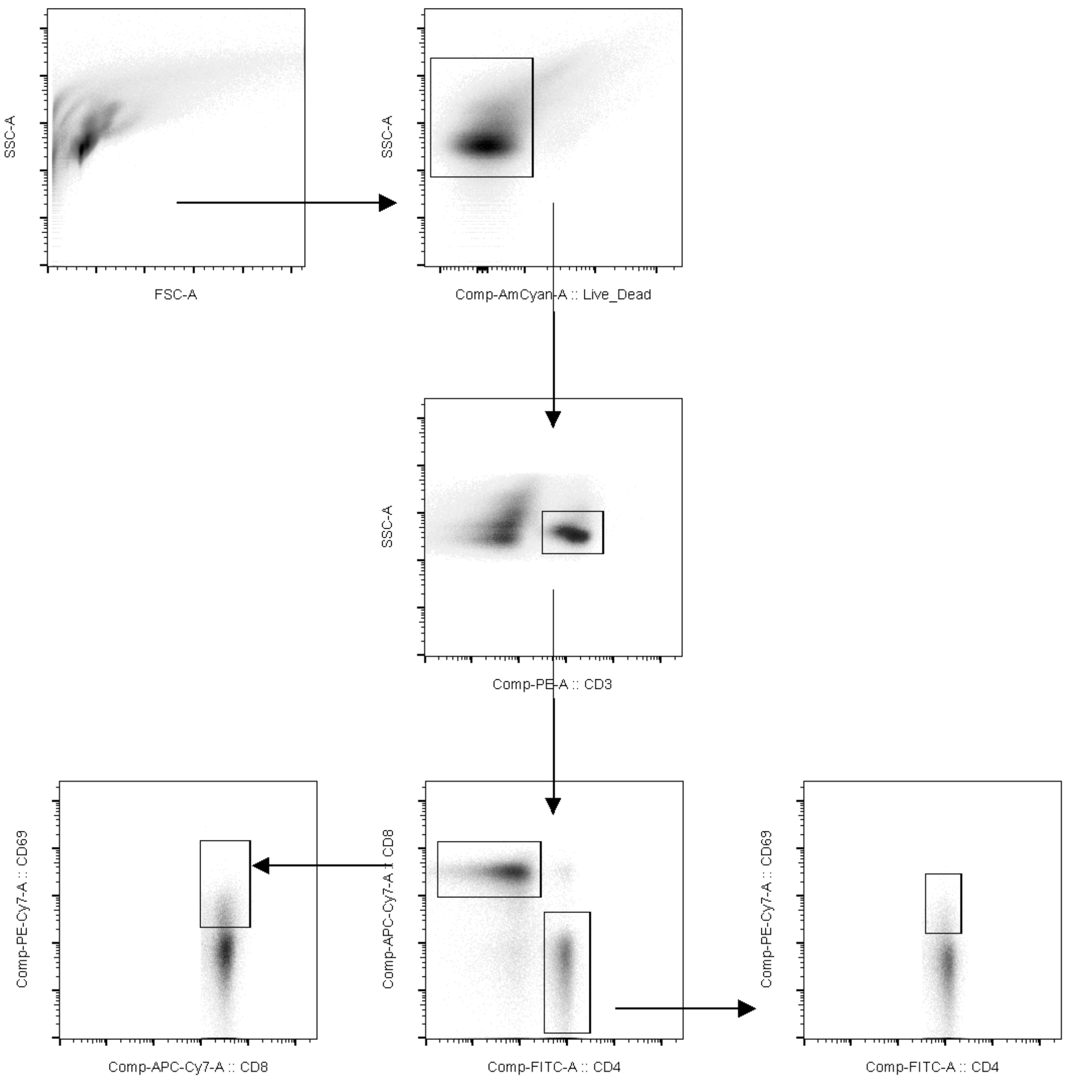

Supplemental Figure 2. Analysis of GR-1$^+$CD11b$^+$ subsets in lung parenchyma. After exclusion of dead cells, the GR-1$^+$ cells, within the CD11b$^+$ subset, were analyzed according to SSC size (high or low). Next both subsets were analyzed for MHC-II and CD11b expression.

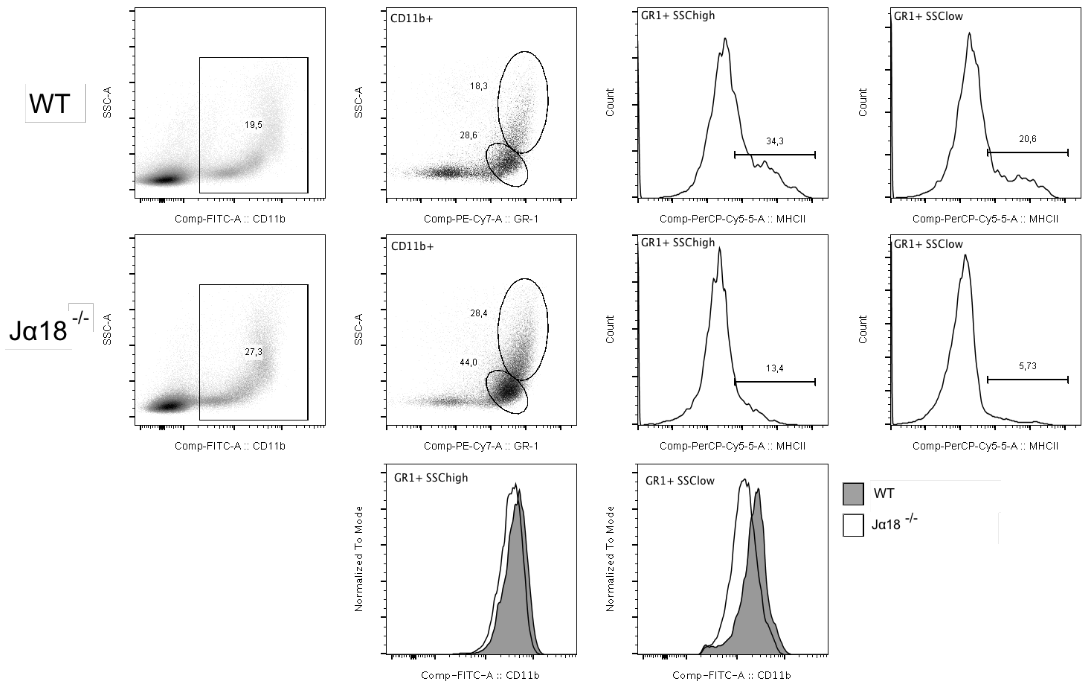

Supplement: Supplementary Materials — Supplemental Figure 1: analysis of CD4 and CD8 T lymphocytes in lung parenchyma. After exclusion of dead cells, the CD3+ cells were analyzed for CD4 or CD8 expression and the frequency of CD69-expressing cells was determined within these populations. Supplemental Figure 2: analysis of GR-1+CD11b+ subsets in lung parenchyma. After exclusion of dead cells, the GR-1+ cells, within the CD11b+ subset, were analyzed according to SSC size (high or low). Next, both subsets were analyzed for MHC-II and CD11b expression. [file 6673722.f1.pdf]
